# Toxicological Assessment of ITER-Like Tungsten Nanoparticles Using an In Vitro 3D Human Airway Epithelium Model

**DOI:** 10.3390/nano9101374

**Published:** 2019-09-25

**Authors:** Isabelle George, Chiara Uboldi, Elodie Bernard, Marcos Sanles Sobrido, Sarah Dine, Agnès Hagège, Dominique Vrel, Nathalie Herlin, Jerome Rose, Thierry Orsière, Christian Grisolia, Bernard Rousseau, Véronique Malard

**Affiliations:** 1 CEA, SCBM, Université Paris Saclay, 91191 Gif-sur-Yvette, France; isabellegeorge87@gmail.com (I.G.); bernard.rousseau@cea.fr (B.R.); 2 Aix Marseille Univ, Avignon Université, CNRS, IRD, IMBE, 13005 Marseille, France; chiara.uboldi@imbe.fr (C.U.); thierry.orsiere@imbe.fr (T.O.); 3 CEA, IRFM, F-13108 Saint Paul lez Durance, France; elodie.bernard@cea.fr (E.B.); christian.grisolia@cea.fr (C.G.); 4 CNRS, Aix Marseille Univ, IRD, INRA, Coll France, CEREGE, 13545, Aix en Provence, France; msanles@irec.cat (M.S.S.); rose@cerege.fr (J.R.); 5 Université Paris 13, Sorbonne Paris Cité; Laboratoire des Sciences des Procédés et des Matériaux, UPR 3407-CNRS, 99 avenue J.-B. Clément 93430 Villetaneuse, France; sarah.dine@lspm.cnrs.fr (S.D.); dominique.vrel@lspm.cnrs.fr (D.V.); 6 Université de Lyon, CNRS, Université Claude Bernard Lyon I, Institute of Analytical Sciences (ISA), UMR 5280, 5, rue de la Doua, 69100 Villeurbanne, France; Agnes.HAGEGE@isa-lyon.fr; 7 NIMBE, IRAMIS, Université Paris Saclay, 91191 Gif sur Yvette CEDEX, France; nathalie.herlin@cea.fr; 8 Aix Marseille Univ, CEA, CNRS, BIAM, UMR7265, EIPM, F-13108 Saint Paul-Lez-Durance, France

**Keywords:** nanoparticles, tungsten, lung, MucilAir™, acute toxicity

## Abstract

The International Thermonuclear Experimental Reactor (ITER) is an international project aimed at the production of carbon-free energy through the use of thermonuclear fusion. During ITER operation, in case of a loss-of-vacuum-accident, tungsten nanoparticles (W-NPs) could potentially be released into the environment and induce occupational exposure via inhalation. W-NPs toxicity was evaluated on MucilAir™, a 3D in vitro cell model of the human airway epithelium. MucilAir™ was exposed for 24 h to metallic ITER-like milled W-NPs, tungstate (WO_4_^2−^) and tungsten carbide cobalt particles alloy (WC-Co). Cytotoxicity and its reversibility were assessed using a kinetic mode up to 28 days after exposure. Epithelial tightness, metabolic activity and interleukin-8 release were also evaluated. Electron microscopy was performed to determine any morphological modification, while mass spectrometry allowed the quantification of W-NPs internalization and of W transfer through the MucilAir™. Our results underlined a decrease in barrier integrity, no effect on metabolic activity or cell viability and a transient increase in IL-8 secretion after exposure to ITER-like milled W-NPs. These effects were associated with W-transfer through the epithelium, but not with intracellular accumulation. We have shown that, under our experimental conditions, ITER-like milled W-NPs have a minor impact on the MucilAir™ in vitro model.

## 1. Introduction

The International Thermonuclear Experimental Reactor (ITER) project is the most important ongoing venture aimed at demonstrating the feasibility of exploiting thermonuclear fusion as an unlimited carbon-free source of energy. One of the main components of the tokamak reactor, the divertor, will be made of tungsten (W). Due to its physical properties, such as robustness and the elevated melting point, W has been selected as the main suitable plasma facing material in tokamaks and future nuclear fusion reactors [1]. Nevertheless, the plasma-wall interaction processes will erode the divertor, leading to the detachment of small tritiated W particles ranging from tens of nanometers to tens of micrometers. To prevent any potential contamination into the environment and/or exposure to workers supervising the tokamak cleaning operations, high efficiency particulate air (HEPA) filters will be used. Nonetheless, those filters have a low retention capability for particles in the 100–500 nm range [2]. In case of a breakdown of the first protection barrier (loss-of-vacuum-accident, LOVA), particles could be accidentally inhaled and this might be harmful for the exposed populations. The consequences, if such activated particles are released into the environment, are unidentified as W-NPs hazard remains largely unknown. It is thus important to assess the impact of W-NPs on lungs in case of accidental inhalation before studying tritiated W-NPs toxicity.

So far, the toxicity of particles containing W was mainly studied in the form of tungsten carbide cobalt alloy (WC-Co) that, due to its hardness, is used in the production of cutting tools and wear-resistant surfaces. Exposure to WC-Co is associated with increased risk of lung cancer [3] and the material is classified as probably carcinogenic to humans (group 2A) by the International Agency for Research on Cancer [4]. The mechanisms governing its genotoxicity can be diverse: Clastogenic and aneuploidogenic events, as well as the generation of reactive oxygen species (ROS) by the particles themselves or via a Fenton-like reaction [5], the harmfulness of WC-Co being related to the presence of cobalt ions release that enhance ROS production [6].

Other potential toxic W forms have been studied. For example, the soluble sodium tungstate has been shown to be non-toxic to hepatocytic cell lines since ATP levels remained generally unchanged following exposure up to 300 μg/mL [7]. Nevertheless, it was shown to increase apoptosis in human peripheral blood lymphocytes [8]. Large particles (27 μm) of metallic tungsten displayed poor to no toxicity on a rat liver cell line [9]. In contrast, small metallic W particles (2.9 μm and 4.3 µm) induced, in a mouse macrophage cell line, a ROS production greater than particles larger than 10 μm [10]. The more severe toxic potential of nanosized particles compared to micrometric ones [11,12] was further confirmed using WC-Co [13] and W particles [14]. In a human-derived bronchial cell line the cytotoxicity of nanosized WC-Co was high compared to that of microparticulated ones and it was strictly related to the internalization rate and pathway [13]. Moreover, in immortalized human-derived alveolar cells, cytotoxicity was observed following exposure to nanometric but not to microsized W metal particles [14].

From a human pathophysiological perspective, after entering into the body through ingestion or inhalation, W might translocate into the blood and then circulate throughout the whole body. Typically, W (as WO_4_^2−^) is rapidly excreted, but some remains in kidney, liver, spleen and bone [15]. The Occupational Safety and Health Administration (OSHA) has established exposure limits up to 5 mg/m^3^ for the insoluble W compounds and 1 mg/m^3^ for the soluble ones used in construction and shipyard industries. Although these data suggest that W metal and its chemical derivatives exhibit low toxicity but a significant oxidative stress on human cells, as recently reviewed [16] little information regarding the effect of W-NPs is currently available.

In the frame of refining, reducing and replacing animal experimentation, alternative in vitro methods have been developed during the last years. There is a clear need to circumvent the time-consuming, cost-expensive and ethically questionable in vivo studies. Currently, most of the in vitro studies are carried out on cell lines and under submerged experimental conditions. However, these exposure conditions seem to have little physiological relevance, especially when related to the lung compartment, which is rather exposed to air-dispersed particles [17]. Furthermore, in vitro 2D cultures generally fail to reconstitute the in vivo microenvironment, and it is also known that cancer cell lines show a too high metabolic activity [18]. On the other hand, human-derived organotypic 3D airway models offer much more reliability: They are fully differentiated respiratory epithelia, they represent functional models displaying metabolic activity, mucus production and cilia beating, they allow air–liquid exposure that more closely resembles the in vivo conditions [19,20].

In this study, we have thus chosen to use the MucilAir™ tissue model characterized by morphology and functions comparable to those of a human epithelium. Obtained from biopsies, MucilAir™ has a lifespan of one year, making it suitable for long-term in vitro studies. It also mimics accidental exposure to a hazard and allows kinetics to be monitored for several weeks to determine short- and long-term toxic effects and their reversibility. Furthermore, the use of such a model represents a valuable in vitro tool to obtain preliminary results on pulmonary absorption of both dissolved and particulate W, avoiding the use of animal experimentation.

Until now, there are no data on human accidental exposure to ITER particles since the reactor is still under construction. Nevertheless, there is a strong need of such preventive information. Therefore, the aim of this study was the in vitro evaluation of the toxicity and the lung absorption of non-tritiated ITER-like milled W-NPs. Since the ITER tokamak is not yet operating, the W-NPs used in this study have been bench synthesized and characterized [21,22]. We have investigated the behavior and the toxic potential of ITER-like W-NPs produced by planetary milling and we have compared them to particulate tungsten carbide alloy doped with cobalt (WC-Co) and to tungstate (WO_4_^2−^) a non-particulate form to compare the speciation of W-NPs in lung. To simulate accidental occupational exposure, MucilAir™ tissues were exposed for 24 h. Various toxicity parameters were monitored up to 28 days post-exposure: Trans-epithelial electrical resistance, cellular metabolism and a pro-inflammatory response. Together, these parameters should be useful to investigate the mechanisms currently thought playing a role in the interaction between particles and lung, such as the role of pulmonary mucus or the rate of particles transepithelial translocation.

## 2. Materials and Methods

### 2.1. Reagents

Soluble tungsten (W; ref 356697) was purchased from Sigma Aldrich (St. Quentin Fallavier, France). Nanostructured WC/6Co powder (WC-Co; ref 74N-062706; purity 99.9%, agglomerates of nanosized particles 40–80 nm) was purchased from Inframat Advanced Materials (Manchester, CT, USA).

### 2.2. Milled Tungsten Nanoparticles (W-NPs)

ITER-like W-NPs have been produced by high energy planetary ball milling with tungsten carbide balls and a jar, a ball-to-powder ratio (BPR) of 40:1 and at a velocity of 350 RPM for a duration of 14 h in ethanol, as previously described [21]. Commercial W, showing 99.9% of purity and particle size 3–12 µm, was used as raw powder material (Alfa Aesar, Karlsruhe, Germany). Milled W-NPs and WC-Co solutions were prepared as described elsewhere [22]. Briefly, particles were suspended in Tris buffer (5 mM, pH 8.5) and sonicated using an ultrasound tip (Sonicator vibracell 72434, Amplitude 40%, 15 min). Large particles were removed by filtration through a 0.45 µm syringe filter; the solutions were then centrifuged and the supernatant containing very small NPs was discarded. The pellet was resuspended in Tris buffer, sonicated again and stored at −20 °C. The amount of W was assessed by inductively coupled plasma mass spectrometry (ICP-MS). The protocol enabled us to produce milled W-NPs suspension with a monodisperse size distribution [22]. A detailed characterization of powders and suspensions has been previously described [21,22,23].

W-NPs suspensions in Tris buffer diluted at 10 µg/cm^2^ were characterized by transmission and scanning electron microscopy (TEM and SEM, respectively) and by dynamic light scattering (Zetasizer nano ZS, Malvern Instruments, Orsay, France), as previously described [24].

### 2.3. Cell Culture and Cellular Morphology

The fully differentiated primary human epithelial MucilAir™ model was purchased from Epithelix Sarl (Geneva, Switzerland). The MucilAir™ cultures used in this study originated from primary human cells isolated from the human nasal cavity of a pool of human non-smokers donors without respiratory pathologies. Signed informed consent and ethical approval were obtained by the supplier. All batches of MucilAir™ were tested negative by the supplier for mycoplasma, human immunodeficiency virus 1 (HIV-1), human immunodeficiency virus 2 (HIV-2) as well as hepatitis B and C.

**Figure 1:**
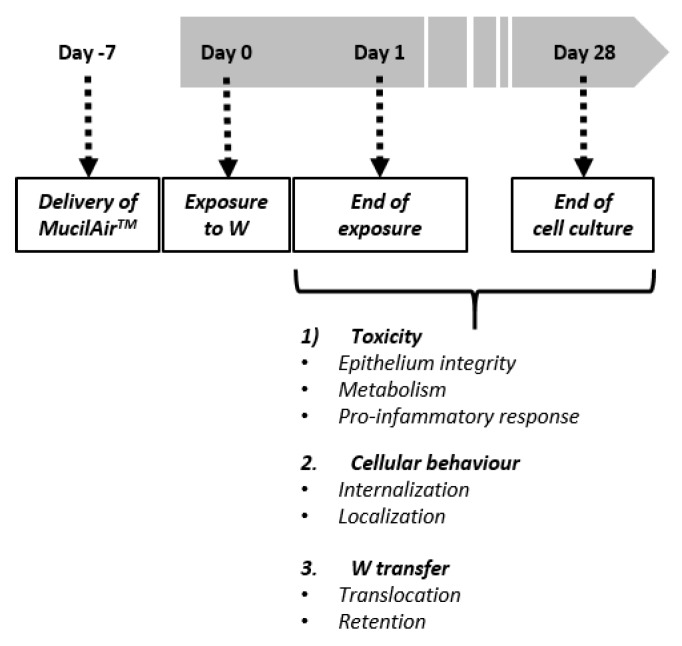
Experimental design. MucilAir™ was kept in culture for one week before exposure to tungsten nanoparticles (W-NPs), tungsten carbide cobalt particles alloy (WC-Co) and WO_4_^2−^. After treatment (24 h), uninternalized W was removed and cells were further cultured for 28 days. During this time several parameters were evaluated at regular intervals to monitor W toxicity, its internalization and intracellular localization, as well as its translocation from the apical to the basolateral compartment.

MucilAir™ models are characterized by a pseudostratified columnar epithelium presenting beating cilia and mucus production. The MucilAir™ model mimics the upper respiratory tract structure of the human lung, including basal, goblet and ciliated cells. Cell culturing was done according to the supplier’s instructions. Concisely, cells were kept at the air–liquid interface (ALI) in 24-well Transwell inserts of 6.5 mm diameter and 0.4 μm pore size (Corning, St. Quentin Fallavier, France). The epithelium was cultivated with MucilAir™ serum-free culture medium (Epithelix Sarl; Geneva, Switzerland) on the basal side. Cells were maintained at 37 °C and 5% CO_2_ for a period up to five weeks (one week before the exposure to W, up to four weeks after exposure; see Figure 1). The basolateral cell culture medium was changed twice a week, while the apical side was washed once a week with a sterile saline solution (NaCl 0.9%, CaCl_2_ 1.25 mM and Hepes 10 mM). By renewing the basolateral medium we were able to preserve the tissue homeostasis, while by washing the apical compartment we removed mucus, surface dead cells and non-internalized ITER-like milled W-NPs or WC-Co. Cellular morphology was assessed using optical microscopy twice a week.

### 2.4. Exposure Protocol

One week after delivery, the MucilAir™ tissues were exposed to 10, 20 and 50 µg/cm^2^ (respectively 110, 220 and 550 µg/mL) of milled W-NPs for 24 h (Figure 1). W-NPs stock solutions were diluted in saline solution and 30 µL of the particles suspension were applied on the apical compartment. Basolateral medium was collected and replaced the first time at the end of the 24 h exposure period, then twice a week during four weeks (28 days post treatment). At the end of the 24 h treatment period the apical side was washed and the mucus collected, then the procedure was repeated twice a week for four weeks after exposure, as described in Figure 1. WC-Co was used as a toxicity control, and WO_4_^2−^ as a positive control for studying W transfer through the epithelium. These controls were applied in the same manner, at the fixed concentration of 10 µg/cm^2^ (110 µg/mL). Negative controls were also performed by exposing cells to saline solution only.

### 2.5. Transmission Electron Microscopy (TEM) on MucilAir™

The cells were washed three times in 0.1 M cacodylate buffer, fixed in 2.5% (*v*/*v*) glutaraldehyde diluted in 0.1 M cacodylate buffer (pH 7.4) and post-fixed in 2% (*v*/*v*) osmium tetroxide. The cells were then dehydrated in a gradient of alcohol solutions and embedded in EMbed 812 kit (Electron Microscopy Sciences; Hatfield, PA, USA). Ultrathin sections (60–70 nm) were not counterstained to optimize their observation with a JEOL JEM1400 electron microscope (JEOL; Tokyo, Japan) at 80 kV. Images were obtained with a Megaview III camera and iTEM Five software (Soft Imaging System; Münster, Germany).

### 2.6. Epithelial Integrity (TEER Measurement)

After W exposure, and twice a week up to 28 days, the tightness of the monolayer was determined by transepithelial electric resistance (TEER) measurement using a STX2 electrode (World Precision Instruments; Hertfordshire, United Kingdom) and the electronic circuit of the EVOM Epithelial Voltohmmeter (World Precision Instruments; Hertfordshire, United Kingdom). 200 µL of saline solution (0.9% NaCl, 1.25 mM CaCl_2_ and 10 mM Hepes) were added onto the apical surface and removed immediately after measurement. To calculate the actual TEER value of each sample, the mean resistance of a cell-free Transwell filter was subtracted from the resistance measured across each MucilAir™ epithelium.

### 2.7. Cell Viability

At the end of the exposure (24 h) and at the end of the culture period (28 days), cells were trypsinized and collected before being pelleted by centrifugation. After discarding the supernatant, cells were resuspended in fresh medium. Cell viability was assessed by Trypan Blue counter staining using the Cedex automated cell counting system (Roche; Basel, Switzerland). Measurements of viable and dead cells were done on two different samples, ten times each sample, to determine the percentage and the number of viable cells.

### 2.8. Metabolic Activity

To measure the cellular metabolism we performed the resazurin assay (Sigma-Aldrich; Saint-Quentin Fallavier, France). This test is based on the measurement of the activity of mitochondrial reductase that catalyzes the reduction of the non-fluorescent substrate resazurin into the fluorescent resorufin by mitochondrial reductases.

The MucilAir™ inserts were transferred in a new 24 wells plate containing 6 µM resazurin in saline solution. Resazurin solution (200 µL) was also applied on the apical surface and the plate was incubated for 1 h at 37 °C and 5% CO_2_. Then, 100 µL of the apical solution were transferred in a 96 wells plate and the fluorescence of the transformed product was measured (excitation filter = 544 nm and emission filter = 590 nm). The MucilAir™ inserts were then re-transferred in a new 24 wells plate containing fresh MucilAir™ culture medium (700 µL per well). The remaining apical solution was removed without unsettling the epithelium and the inserts were put back into the incubator. The measurements were performed in duplicate on three to five inserts.

### 2.9. Interleukin-8 Assay

To estimate the pro-inflammatory response induced by exposure to W, we quantified the levels of interleukin-8 (IL-8). Briefly, basolateral media were removed and centrifuged at 10,000× *g* (10 min at 4 °C) to eliminate cellular debris and NPs, then stored at −20 °C. IL-8 was quantified using an ELISA kit (DY208, R & D Systems; Minneapolis, MN, USA). The optical density was measured at 450 nm with a microplate photometer Elx800 (Biotek; Colmar, France). The measurements were performed in duplicate on three to five inserts.

### 2.10. Tungsten Extracellular and Intracellular Quantification

To estimate the cellular uptake of W and the transfer through the lung epithelium after treatment, apical and basolateral media were removed and stored at −20 °C. Cells were also trypsinized and collected. Tungsten was recovered by dissolution using 80 volumes of 30% H_2_O_2_ and four volumes of NH_4_OH within 12 h at room temperature. Quantification was performed by inductively coupled plasma mass spectrometry (ICP-MS; 7700, Agilent Technologies; Santa Clara, CA, USA) at m/z = 182, 184 and 186 after dilution in ultrapure water. The standard curve (0.1 to 10 ng/g of W) was prepared from the plasmaCAL standard (ref 140-050-741 SCPscience; Courtaboeuf, France). The detection limits given by the MassHunter software were 0.007 ng/g on ^182^W, 0.001 ng/g on ^184^W and 0.002 ng/g on ^186^W, and calculated at 0.02 ng/g. Each sample was measured in triplicate and a blank was done after each sample. The measurements were performed on three to six samples for apical and basolateral media and on two cellular samples.

### 2.11. Statistical Analysis

Results are expressed as the mean ± SEM or mean ± SD. Depending on the time points the number of samples analyzed (*n*) was variable because some inserts were progressively used to perform ICP-MS or TEM analysis. For the statistical evaluation, a one-way ANOVA followed by Dunnett’s multiple comparisons test were performed using GraphPad Prism version 8 (GraphPad Software; La Jolla, CA, USA).

## 3. Results

### 3.1. W-NPs Characterization

**Figure 2:**
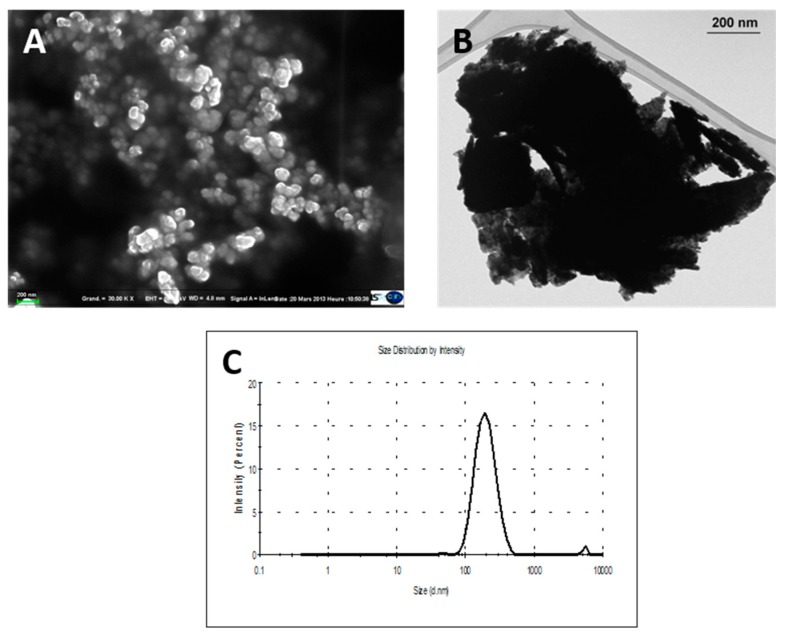
Morphometric analysis and International Thermonuclear Experimental Reactor (ITER)-like milled W-NPs size determination. W-NPs solutions resulted polydispersed, as shown by SEM (**A**) and TEM (**B**). The dispersion of particles in Tris (pH 8.5) was analyzed by dynamic light scattering (DLS; **C**). The mean intensity ± standard deviation (SD) corresponded to 202 ± 68 nm, and polydispersity index = 0.307 ± 0.02 (*n* = 3).

A detailed description of the physicochemical properties of ITER-like milled W-NPs has been previously published [21,22]. Scanning electron microscopy (SEM; Figure 2A) and transmission electron microscopy (TEM; Figure 2B) micrographs showed that milled W-NPs exhibited a polyhedral structure with a size range of 50–100 nm. W-NPs size distribution (Figure 2C) was further determined by dynamic light scattering (DLS) after thawing of the particle stock solutions [22]. The main intensity peak (98.5% intensity) corresponded to a mean hydrodynamic size diameter of 202 ± 68 nm, and the polydispersity index was 0.31 ± 0.02 (*n* = 3).

### 3.2. MucilAir™: Tissue Characterization and Morphological Modifications Upon Exposure to W

**Figure 3:**
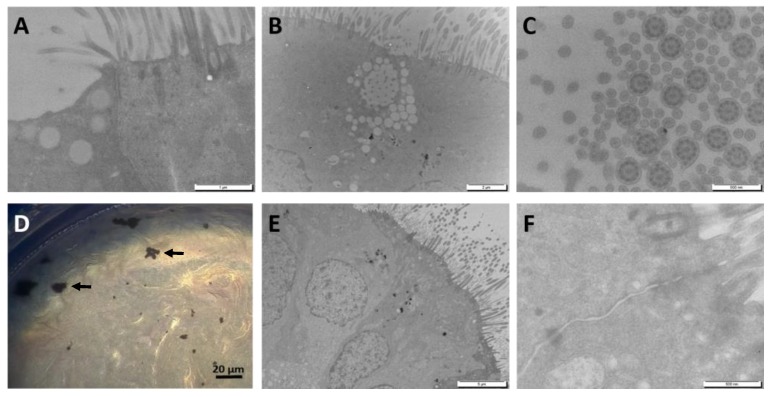
Optical microscopy and TEM observation of MucilAir™ before and after exposure to milled W-NPs. The morphology of MucilAir™ was observed before (**A**–**C**) and after (**D**–**F**) exposure to 50 µg/cm^2^ milled W-NPs. Tight junctions, ciliated cells and mucous cells, presenting several mucous vesicles, were observed (**A**,**B**; TEM). A cross sectional image (**C**; TEM) showed that MucilAir™ cilia have a typical ultrastructure with axonemes, dynein arms and central tubules. At the end of the exposure (**D**; optical microscopy), small aggregates/agglomerates of W-NPs were onto the borders of the insert. At day 28 post exposure (**E**,**F**; TEM), the confluency of the tissue is maintained, as well as tight junctions. Size bars: A = 1 µm; B = 2 µm; C = 500 nm; D = 20 µm; E = 5 µm; E = 5 µm, F = 500 nm.

Upon receipt, MucilAir™ was maintained in culture for at least one week before performing the experiments. The morphology of the tissues was evaluated using electron microscopy (Figure 3). An indicator of the integrity of the epithelium is the typical ultra-structure of the human airway epithelium, such as the presence of tight junctions, as well as of ciliated, basal and mucus cells, as observed in our samples (Figure 3A–C). At the end of the exposure period, aggregated W-NPs tended to accumulate onto the borders of the insert (Figure 3D). Twenty-eight days after exposure to W-NPs the confluency of the tissue and the tight junctions were preserved (Figure 3E,F).

### 3.3. Cell Viability and Metabolic Activity

Cells from MucilAir™ were collected by trypsinization at day 1 and day 28 after exposure to milled W-NPs, WC-Co and WO_4_^2−^. Viable cells were determined by the trypan blue method using an automated cell counter. Compared to the negative control (C neg), as shown in Table 1, cell viability after exposure to 10, 20 and 50 µg/cm^2^ of W-NPs and to 10 µg/cm^2^ of WO_4_^2−^ and WC-Co was not significantly modified.

We also evaluated the physiological status of MucilAir™ by analyzing their metabolic activity by a resazurin test (Table 2). The evaluation was performed before (day 0) treating MucilAir™, after 24 h (day 1) and at day 7, 14, 21 and 28 post exposure to milled W-NPs, WO_4_^2−^ and WC-Co. Compared to C neg, ITER-like milled W-NPs did not significantly impair the metabolic activity of MucilAir™, with the exception of a slight significant raise (*p* < 0.05) at day 14 after incubation with 20 µg/cm^2^ ITER-like milled W-NPs. By contrast, WC-Co exerted a statistically significant (*p* < 0.001) reduction in cell viability at day 1 and day 7, which was fully restored from day 14 onwards. No modification was observed in MucilAir™ exposed to WO4^2−^.

### 3.4. IL-8 Release

The pro-inflammatory cytokine IL-8 was quantified in the basolateral medium upon MucilAir™ exposure and data are presented in Table 3. IL-8 secretion was enhanced by milled W-NPs from day 1 to day 28, mainly at day 4, independently of the tested concentration (10, 20 and 50 µg/cm^2^). In some cases the IL-8 levels were statistically significant (*p* < 0.05 and *p* < 0.01) compared to C neg.

WC-Co enhanced a statistically significant IL-8 production at day 1 and day 4 (*p* < 0.001), while it decreased from day 7 to 14, to finally regain at day 21 and 28 post incubation. In comparison with W-NPs, IL-8 secretion was found 1.5 times higher at day 4. WO_4_^2−^ induced IL-8 secretion but was statistically significant (*p* < 0.01) only at day 4.

### 3.5. Epithelial Integrity: TEER Measurements

In order to verify that MucilAir™ was ready to be exposed to ITER-like milled W-NPs (10, 20 and 50 µg/cm^2^) and to WC-Co (10 µg/cm^2^) and WO_4_^2−^ (10 µg/cm^2^), TEER was checked before starting the experiments. The measured values were always above 330 ohms/cm^2^.

**Figure 4:**
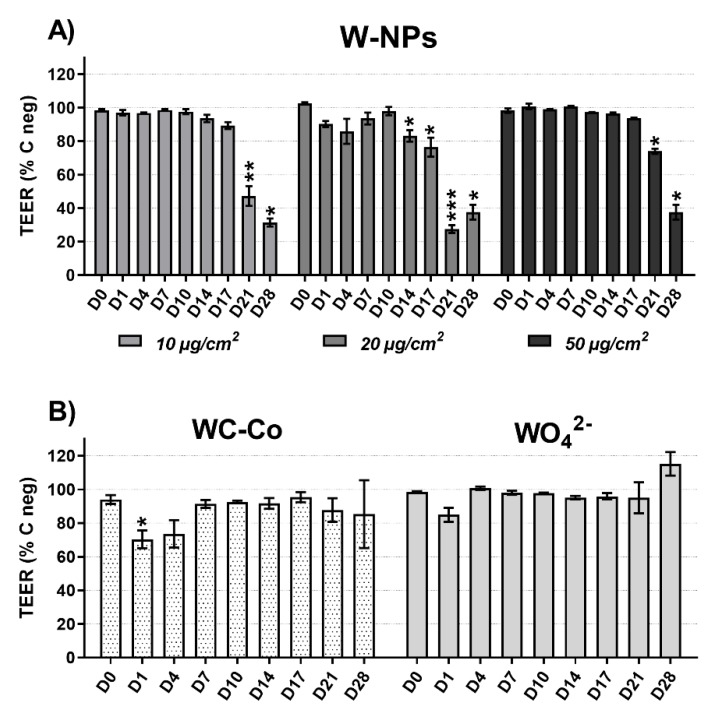
Effect of milled W exposure on transepithelial electric resistance (TEER): (**A**) W-NPs, (**B**) WC-Co and WO_4_^2−^. The measurements were performed at the end of the 24 h exposure (day 1), and then at day 4, 7, 10, 14, 17, 21 and 28 post exposure. The results are expressed as a percentage of TEER compared to C neg ± SEM of three to six individual inserts. Statistically significant differences from C neg were determined by a two-way ANOVA followed by Dunnett’s multiple comparisons test: * *p* < 0.05, ** *p* < 0.01 and *** *p* < 0.001.

As shown in Figure 4A, 10 µg/cm^2^ W-NPs did not cause any TEER change until day 17 post-exposure, whereas at day 21 and day 28 the damage was significant (*p* < 0.01) and corresponded to a 52% and 62% TEER decrease, respectively. The exposure to 20 µg/cm^2^ W-NPs had no effects until day 10 and from day 14 onwards TEER reduction was statistically significant: 17% (*p* < 0.05) and 24% (*p* < 0.05) at day 14 and 17, and 73% (*p* < 0.001) and 55% at day 21 and 28, respectively. Finally, 50 µg/cm^2^ W-NPs were affecting the MucilAir™ integrity at day 21 and 28 (26% and 55% TEER reduction, respectively).

Neither WC-Co nor WO_4_^2−^ induced statistically significant TEER impairment in MucilAir™ (Figure 4B), although WC-Co exerted a 30% and 27% TEER reduction at day 1 (*p* < 0.05) and day 4, respectively, and WO_4_^2−^ caused a 15% TEER decrease at day 1 post-exposure.

### 3.6. Transepithelial Passage of W and Its Intracellular Accumulation

In order to evaluate the absorption of W by the epithelial pulmonary MucilAir™ tissue, the extracellular and the intracellular amount of W were quantified from day 1 to day 28 post exposure using inductively coupled mass spectrometry (ICP-MS).

**Figure 5:**
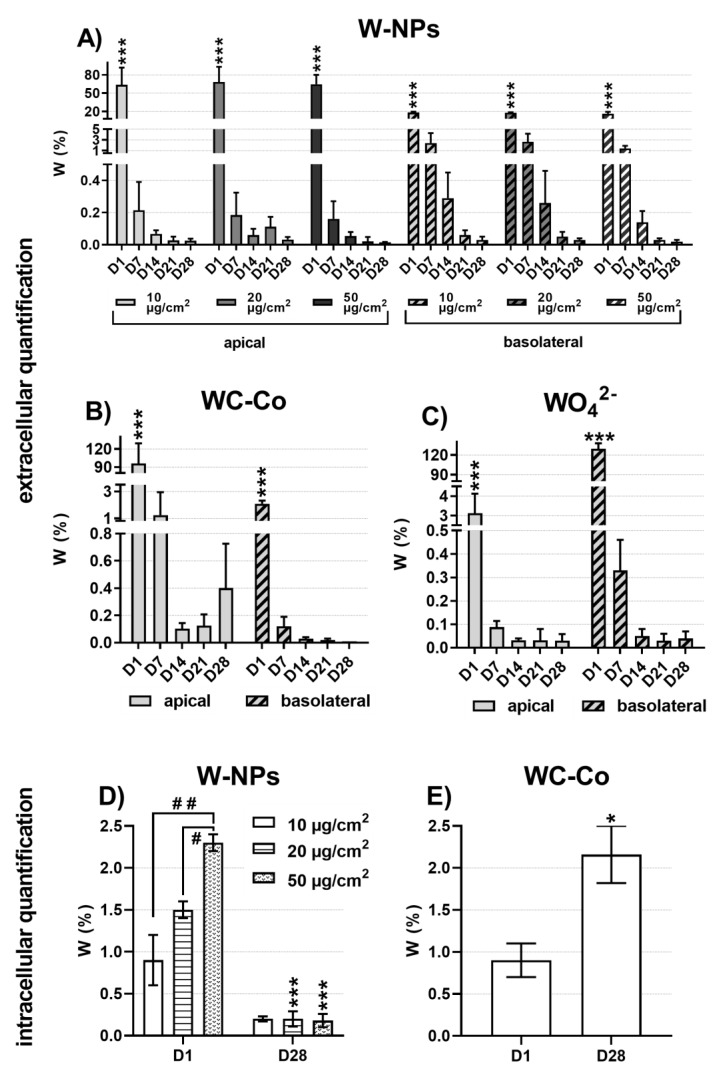
Transepithelial passage of W and cellular accumulation: extracellular quantification of (**A**) W-NPs, (**B**) WC-Co, (**C**) WO_4_^2−^ and intracellular quantification of (**D**) W-NPs, (**E**) WC-Co. W content was assessed in both apical and basal media of MucilAir™, as well as in cells, using inductively coupled plasma mass spectrometry (ICP-MS) after mineralization of the samples. The results are expressed as W% in the exposure solution ± SD at the end of the 24 h treatment (day 1), and at day 7, 14, 21 and 28 post exposure. Statistically significant differences from C neg were determined by a one-way ANOVA followed by Dunnett’s multiple comparisons test: * *p* < 0.05 and *** *p* < 0.001.

The extracellular quantification (mean % W ± SD) was performed on mineralized apical and basolateral cell culture media (Figure 5A–C). At the end of exposure to 10, 20 and 50 µg/cm^2^ of milled W-NPs (Figure 5A), a significant fraction of W, ranging from 64% to 68%, was present in the apical medium while a smaller amount (16%–18%) was able to translocate in the basolateral compartment.

From day 7 to day 28 post exposure to milled W-NPs (Figure 5A) the quantity of W still present in the apical compartment severely and rapidly decreased compared to day 1, ranging from 0.2% ± 0.1% at day 7 to 0.01% ± 0.01% at day 28 (*p* < 0.001). A similar behavior was observed in the basolateral samples: 2.7% ± 0.5% was the W amount quantified at day 7, whereas at day 28 the amount of W corresponded to 0.02% ± 0.01% (*p* < 0.001).

The extracellular quantification of WC-Co (Figure 5B) and WO_4_^2−^ (Figure 5C) showed a different behavior in comparison to W-NPs. The main content of WC-Co at day 1 was found at the apical side 94.1% ± 33.2% while it decreased between 1.2% ± 0.7% and 0.1% ± 0.04% at the next time points (Figure 5B). The basolateral content was very low compared to the apical one (2.1% ± 0.2% at day 1) while a significant decrease (*p* < 0.001) was observed during days 7–28 (0.1% ± 0.1% to 0.01% ± 0.01%). After exposure to the soluble WO_4_^2−^, W was more present in the basolateral (129.7% ± 8.3%) than in the apical compartment (3.1% ± 1.0%) at day 1 (Figure 5C). From day 7 onwards the detectable W ranged from 0.09% ± 0.03% to 0.03% ± 0.01% in the apical medium and from 0.3% ± 0.1% to 0.03% ± 0.03% in the basolateral one (Figure 5C).

Lastly, to quantify the intracellular level of W upon MucilAir™ exposure to W-NPs (Figure 5D) and WC-Co (Figure 5E), ICP-MS was performed on mineralized cells that were previously trypsinized. A dose-related effect was observed at day 1 in cells exposed to W-NPs (Figure 5D), when the amount of W internalized by MucilAir™ corresponded to 0.9% ± 0.3%, 1.5% ± 0.1% and 2.3% ± 0.1% upon exposure to, respectively, 10, 20 and 50 µg/cm^2^ W-NPs. On the other hand, at day 28 post exposure a significantly (*p* < 0.01) reduced uptake of W took place, whose values were comprised between 0.2% ± 0.03% and 0.2% ± 0.09%. WC-Co intracellular quantification (Figure 5E) showed an opposite behavior: The amount of W was higher at day 28 (2.2% ± 0.3%) than at day 1 (0.9% ± 0.2%).

## 4. Discussion

During operation of fusion power plants like ITER the divertor is supposed to interact with the plasma generating thus tritiated tungsten dusts that, in case of LOVA, can be released into the environment. The protection of the enclosures is ensured by HEPA filters which have a low efficiency for particles with an aerodynamic diameter of 100–500 nm. For tungsten, due to its high density, this corresponds to particles with a geometric diameter of 20–100 nm. Therefore, in the event of a containment accident, humans could be exposed by inhalation to W-NPs. Since at present there is no conclusive information on the toxic effects potentially exerted by W-NPs, the aim of our study was to evaluate the toxicity of non-tritiated ITER-like W-NPs by applying them to the MucilAir™.

We have thus chosen to perform a single 24 h exposure of MucilAir™ to increasing concentrations of ITER-like milled W-NPs, mimicking thus an accidental exposure that could occur in case of LOVA. To evaluate the reversibility or the accumulation of the effects, several parameters were investigated during the 28 days post-exposure to milled W-NPs, WC-Co and WO_4_^2−^. Our study of the toxic potential of ITER-like milled W-NPs showed that, for concentrations up to 50 µg/cm^2^, neither the viability nor the metabolic activity of MucilAir™ were altered. The pro-inflammatory chemokine, interleukin 8 was chosen since it represents an early indicator of the pro-inflammatory cascade and has been shown to be up-regulated in response to a number of NPs [25]. For all tested concentrations, milled W-NPs transiently enhanced the release of the pro-inflammatory cytokine IL-8 in the basolateral compartment of MucilAir™. Barrier integrity was impaired with no dose-related effects at late time points (day 14 onwards). ICP-MS showed that at the end of exposure (day 1) W was massively present in the apical media and was taken up by MucilAir™ cells in a dose-related manner, while from day 7 to 28 its extracellular and intracellular levels were significantly reduced.

The current literature reports few studies on nanosized W particles and, to our knowledge, only one has been performed on metal W nanoparticles, in particular on the effects of micrometric and nanometric debris derived from projectiles made of heavy W alloys, and compared them to commercially manufactured metal W-NPs [14]. The physico-chemical characterization showed that the tested samples contained metallic W of about 50 nm diameter, which is comparable to our bench prepared ITER-like milled W-NPs. The cytotoxicity was investigated in human-derived A549 lung epithelial cells by colorimetric assay upon 48 h exposure. Results showed that while the micrometric form of W is not toxic on A549 cells at any of the tested concentrations (2.5, 5 and 10 µg/mL), the nanosized elemental W exerted a 3.5–4.5 fold-increased cytotoxicity [14].

Under our experimental conditions we have observed no decreased viability nor altered metabolic activity upon exposure to milled W-NPs, whereas we have observed a slight impairment of the barrier integrity. These results seem even more significant considering that, compared to Machado and co-authors [14], we have applied significant higher ITER-like W-NPs mass: 10, 20 and 50 µg/cm^2^ correspond, respectively, to 110, 220 and 550 µg/mL of milled W-NPs in MucilAir™. This different response to metallic W could be due to the significant difference between an ex vivo multicellular 3D model, such as MucilAir™, and an immortalized cell line, such as A549. Our hypothesis is that the mucous layer present on the surface of MucilAir™ has lowered the interaction between milled W-NPs and lung cells, protecting thus the cells from severe damage. Even if also A549 cells produce surfactant, their exposure to nanoparticles was done in culture medium and not on air–liquid as for MucilAir™. This might result in a less effective mucous protection in A549 cells whose cellular viability was, consequently, impaired. This assumption has already been made in another study on the MucilAir^TM^ model [26].

Whereas currently in the literature almost no reference is available on W nanoparticles, some studies have been conducted on the toxicity of various forms of tungsten such as sodium tungstate (Na_2_WO_4_) and tungsten oxide (WO_3_). Na_2_WO_4_ was reported to increase the frequency of apoptotic human derived peripheral blood lymphocytes (PBL) and to alter their cell cycle regulation at doses comprised between 0.1 and 10 mM and exposure times ranging from 24 to 72 h [8]. Additionally, Na_2_WO_4_ was shown to enhance the carcinogenic properties of human-derived bronchial epithelial BEAS-2B cells: By deregulating genes involved in lung cancer and leukemia control, clones of BEAS-2B exposed to Na_2_WO_4_ were able to increase tumor development in nude mice [27]. Tungsten oxide particles (WO_3_ NP), with an average diameter of 223.9 ± 5.3 nm in complete culture medium were reported to decrease viability and increase membrane damage in a concentration (up to 300 µg/mL) and time (24–48 h) related manner in A549. Moreover, time- and dose-dependent increased intracellular and extracellular oxidative stress markers were measured [28]. In contrast, no toxicity was observed on the MucilAir™ model exposed 24 h to WO_3_ nanoparticles. Upon exposure to doses corresponding to a high level of exposure in vivo, neither viability (lactic dehydrogenase assay), nor the release of pro-inflammatory cytokines (IL-6, IL-8 and MCP1) were affected [26].

In the current literature extensive information is reported on tungsten carbide (WC) and the micrometric tungsten carbide cobalt (WC-Co) alloys. For instance, the metabolic activity and the membrane integrity of immortalized human derived alveolar A549 cells were not altered by WC, while a slight decrease in membrane integrity was observed upon WC-Co exposure (0–33 µg/mL, 72 h) [29]. Moreover, WC-Co exerted a more severe and significant decrease in A549 metabolic activity and membrane integrity compared to the soluble CoCl_2_ and to WC supplemented with CoCl_2_, underlining and confirming thus the importance of the particulate form for the induction of toxicity [29]. The mechanisms of WC-Co toxicity have been attributed to its capability of producing ROS that are known to induce cell damage [30] and inhibit DNA repair mechanisms [31]. The origin of these oxidative species has been attributed either to an intrinsic generation of ROS operated by the particles themselves and to the transfer of electrons from Co to WC, either to a Fenton-like reaction catalyzed by Co-ions released from WC-Co particles [5]. In both cases, ROS generation rapidly occurs and is related to WC-Co internalization. This was demonstrated by comparing the cytogenotoxic effects of WC-Co in three epithelial cell lines, namely human-derived lung A549, renal Caki-1 and hepatic Hep3B cells: ROS WC-Co-dependent production enhanced cellular mortality, DNA damage and cell cycle arrest in Caki-1 and Hep3B cells, while no effects were observed in A549 cells, which not only showed a lower particles uptake but also an ability to release them [32].

Using the trypan blue assay, under our experimental conditions, WC-Co did not induce cytotoxic effects; on the contrary, resazurin assay showed that WC-Co slightly affected the metabolism of MucilAir™. IL-8 release occurred in basolateral compartment of MucilAir™ and it was statistically significant at specific time points (day 1, 4 and 14) compared to W-NPs. Moreover, 10 µg/cm^2^ of WC-Co damaged the MucilAir™ barrier integrity at much earlier time points (day 1 and 4) than W-NPs, whose effects were significant from day 14 onwards.

Our data suggested that the impairment of the metabolic activity is linked, through an adaptive phenomenon, to the epithelial integrity: When a mild alteration of the barrier integrity occurs, in the absence of cytotoxicity or of severe metabolic damage, ionic channels are activated [33] without breaking tight junctions or generating holes in the epithelium. Under our experimental conditions, 28 days after exposure to W-NPs, we observed under electron microscopy intact tight junctions whereas a decrease of TEER occurred. Therefore our hypothesis is that WC-Co and W-NPs induced ion channels activation as a defensive mechanism to eliminate ionic W released over the time into the MucilAir™. We are also suggesting that the level of transiently released pro-inflammatory IL-8 in basolateral compartment of MucilAir™, in the presence of WC-Co and of ITER-like milled W-NPs, was not sufficient to damage the barrier. Although IL-8 secretion was enhanced, it remained significantly lower compared to those measured by Huang and collaborators in MucilAir™ exposed to well-known respiratory irritants [34], indicating thus that the inflammatory potential of W is not severe.

We could explain the W-NPs and the WC-Co results taking into consideration the extracellular and intracellular accumulation of W. The high amount of W measured by ICP-MS in the apical compartment of MucilAir™, as well as its presence in the basolateral medium already at day 1 post exposure, allows us to hypothesize that a rapid transepithelial translocation of W occurred. We could further assume that a fraction of WC-Co remained trapped in the mucous layer and that a slow but continuous W release and internalization took place since at day 28 the intracellular amount of W, in WC-Co exposed MucilAir™, was higher compared to day 1. On the contrary, a dose-related intracellular accumulation of W in tissues exposed to milled W-NPs was detected at day 1 but not at day 28. The lower solubility of WC-Co compared to ITER-like W-NPs has contributed to this opposite behavior. Certainly our data will contribute to define the quantitative absorption or distribution of inhaled W in human lungs.

From the literature it is evident that lung cells are able to internalize metallic nanoparticles, sometimes even in a more efficient manner than their corresponding soluble metal form [24]. Since under our conditions W was taken up by MucilAir™, we tried to understand under which form is W absorbed through the lung epithelium. SEM and TEM studies did not show W-NPs internalization, while ICP-MS showed an intracellular uptake of W; these results suggest thus that a solubilization has occurred in the mucous layer, and are in contrast with a previous study that demonstrated the insolubility of W [16]. Hence, the physico-chemical behavior of the ITER-like milled W-NPs used in this study was investigated [22]. It was detected that in, the very same saline solution we applied to the apical compartment of MucilAir™, 20% of W was solubilized within 24 h and that in the culture medium used in the basolateral compartment the solubilization raised up to 40% [22]. These results clearly indicated that tungsten metal particles could solubilize in biological media at pH 7.5. Tungstate (WO_4_^2−^) is likely the most bioavailable form of tungsten and the main form absorbed through the lung epithelium after exposure to W-NPs. Furthermore, the rate of dissolution and transformation to tungstate will depend of the size of W-NPs, as well as of the pH and the composition of their surrounding environment [16,35]. Our hypothesis is that milled W-NPs remained trapped into the mucous layer of MucilAir™ where they solubilized allowing thus W internalization, as detected by ICP-MS, and suggesting that the mucous layer might protect the cells from particles uptake and from severe cytotoxicity.

## 5. Conclusions

We performed the first acute toxicity study of tungsten metal W-NPs on MucilAir™, a 3D in vitro model of the human airway epithelium, and we have shown that W-NPs had a limited impact in terms of toxic effects, cellular uptake and W transfer through the lung epithelium. MucilAir™ appeared as a valuable experimental tool to further study the toxicity of the tritiated tungsten nanoparticles. The latter ones will be generated during the operation of the ITER fusion reactor. The study of the transfer of tritium through the lung epithelium, as well as the toxicity exerted, may provide preliminary information to biokinetic studies aimed at defining biokinetic lung models for ITER-like tritiated W-NPs, to establish new safety rules and radiation protection approaches.

## Figures and Tables

**Table 1:** Comparison of the viability of MucilAir™ cells at different time points.

		Day 1	Day 28
**C neg**		88.0 ± 0.7	82.7 ± 3.1
**W-NPs**	**10 µg/cm^2^**	77.3 ± 6.7	78.8 ± 1.5
	**20 µg/cm^2^**	79.8 ± 0.5	77.4 ± 2.8
	**50 µg/cm^2^**	85.9 ± 0.4	82.0 ± 0.6
**WC-Co**	**10 µg/cm^2^**	80.5 ± 0.9	81.1 ± 1.1
**WO_4_^2−^**	**10 µg/cm^2^**	87.5 ± 0.9	80.2 ± 0.4

MucilAir™ cells viability was quantified by Trypan blue assay. Cells were trypsinized and collected at the end of the 24 h exposure (day 1) to W-NPs, WC-Co and WO_4_^2−^, and at day 28 post-exposure. No statistically significant differences from the C neg were observed by a one-way ANOVA followed by Dunnett’s multiple comparisons test.

**Table 2:** Viability evaluation of MucilAir™ via the analysis of the metabolic activity: Resazurin assay.

		Day 0	Day 1	Day 7	Day 14	Day 21	Day 28
**C neg**		100.0 ± 6.1%	100.0 ± 1.6	100.0 ± 4.5	100.0 ± 3.9	100.0 ± 4.4	100.0 ± 4.8
**W-NPs**	**10 µg/cm^2^**	106.9 ± 3.8%	97.7 ± 2.1	107.5 ± 5.2	106.2 ± 3.3	93.2 ± 4.1	107.7 ± 2.4
	**20 µg/cm^2^**	102.9 ± 4.8%	103.0 ± 1.8	112.5 ± 3.2	119.6 ± 5.4 *	109.4 ± 4.6	108.1 ± 7.2
	**50 µg/cm^2^**	102.8 ± 3.8%	102.7 ± 2.0	113.2 ± 3.0	110.1 ± 1.5	103.7 ± 3.2	118.8 ± 4.3
**WC-Co**	**10 µg/cm^2^**	99.6 ± 3.9	91.1 ± 1.8 **	82.1 ± 2.7 *	104.2 ± 5.3	102.4 ± 7.4	95.8 ± 8.4
**WO_4_^2−^**	**10 µg/cm^2^**	102.4 ± 5.0	101.9 ± 1.4	114.7 ± 3.3	113.1 ± 5.4	102.9 ± 8.3	100.7 ± 6.5

The metabolic activity of MucilAir™ exposed to W-NPs, WC-Co and WO_4_^2^**^−^** was evaluated by resazurin assay. Analyses were performed before (day 0) and 24 h after exposure (day 1), and then at regular time points (day 7, 14, 21 and 28). Data are expressed as the % of cell viability (mean ± SEM) compared to the respective C neg (untreated control cells). Statistically significant differences from the C neg were determined by one-way ANOVA followed by Dunnett’s multiple comparisons test: * *p* < 0.05, ** *p* < 0.01.

**Table 3:** Quantification of the release of the pro-inflammatory cytokine IL-8 in MucilAir™ basolateral compartments.

		Day 1	Day 4	Day 7	Day 14	Day 21	Day 28
**C neg**		1876 ± 380	2575 ± 87	3415 ± 179	5188 ± 292	4902 ± 258	5162 ± 80
**W-NPs**	**10 µg/cm^2^**	1454 ± 259	4615 ± 742 *	5060 ± 593	6072 ± 247	5567 ± 852	5713 ± 504
	**20 µg/cm^2^**	2473 ± 401	5288 ± 464 **	5261 ± 655 *	6782 ± 213 *	7023 ± 253 *	5661 ± 296
	**50 µg/cm^2^**	2668 ± 313	4681 ± 347 *	5017 ± 406	4799 ± 626	4471 ± 155	5433 ± 341
**WC-Co**	**10 µg/cm^2^**	34112 ± 375 **	6833 ± 265 ***	4673 ± 54	4660 ± 263	6237 ± 326	6016 ± 245
**WO_4_^2−^**	**10 µg/cm^2^**	2215 ± 108	5098 ± 285 ***	4838 ± 343	5707 ± 202	5563 ± 325	5246 ± 315

The amount of IL-8 released by MucilAir™ was quantified in the basolateral compartment by ELISA. Samples of cell culture media were collected at fixed time points ranging from the end of the 24h exposure (day 1) to 28 days post-exposure. Results are expressed as pg/mL. The statistically significant differences from C neg were determined by a one-way ANOVA followed by Dunnett’s multiple comparisons test: * *p* < 0.05, ** *p* <0.01 and *** *p* < 0.001.

## References

[1] Davis J.W., Barabash V.R., Makhankov A., Plochl L., Slattery K.T. (1998). Assessment of tungsten for use in the ITER plasma facing components. J. Nucl. Mater..

[2] Huang S.H., Chen C.W., Kuo Y.M., Lai C.Y., McKay R., Chen C.C. (2013). Factors Affecting Filter Penetration and Quality Factor of Particulate Respirators. Aerosol Air Qual. Res..

[3] Moulin J.J., Wild P., Romazini S., Lasfargues G., Peltier A., Bozec C., Deguerry P., Pellet F., Perdrix A. (1998). Lung Cancer Risk in Hard-Metal Workers. Am. J. Epidemiol..

[4] IARC (2006). Monographs on the Evaluation of Carcinogenic Risks to Humans: Metallic Cobalt Particles.

[5] Moche H., Chevalier D., Vezin H., Claude N., Lorge E., Nesslany F. (2015). Genotoxicity of tungsten carbide–Cobalt (WC–Co) nanoparticles in vitro: Mechanisms-of-action studies. Mutat. Res. Genet. Toxicol. Environ. Mutagenesis.

[6] Lison D., Carbonnelle P., Lauwerys R., Mollo L., Fubini B. (1995). Physicochemical Mechanism of the Interaction between Cobalt Metal and Carbide Particles To Generate Toxic Activated Oxygen Species. Chem. Res. Toxicol..

[7] Johnson D.R., Ang C., Bednar A.J., Inouye L.S. (2010). Tungsten Effects on Phosphate-Dependent Biochemical Pathways are Species and Liver Cell Line Dependent. Toxicol. Sci..

[8] Osterburg A.R., Robinson C.T., Schwemberger S., Mokashi V., Stockelman M., Babcock G.F. (2010). Sodium tungstate (Na2WO4) exposure increases apoptosis in human peripheral blood lymphocytes. J. Immunotoxicol..

[9] Hussain S.M., Hess K.L., Gearhart J.M., Geiss K.T., Schlager J.J. (2005). In vitro toxicity of nanoparticles in BRL 3A rat liver cells. Toxicol. In Vitro.

[10] Stefaniak A.B., Leonard S.S., Hoover M.D., Virji M.A., Day G.A. (2009). Dissolution and reactive oxygen species generation of inhaled cemented tungsten carbide particles in artificial human lung fluids. J. Phys. Conf. Ser..

[11] Nel A., Xia T., Madler L., Li N. (2006). Toxic potential of materials at the nanolevel. Science.

[12] Xia T., Kovochich M., Brant J., Hotze M., Sempf J., Oberley T., Sioutas C., Yeh J.I., Wiesner M.R., Nel A.E. (2006). Comparison of the abilities of ambient and manufactured nanoparticles to induce cellular toxicity according to an oxidative stress paradigm. Nano Lett..

[13] Armstead A.L., Arena C.B., Li B. (2014). Exploring the potential role of tungsten carbide cobalt (WC-Co) nanoparticle internalization in observed toxicity toward lung epithelial cells in vitro. Toxicol. Appl. Pharmacol..

[14] Machado B.I., Suro R.M., Garza K.M., Murr L.E. (2011). Comparative microstructures and cytotoxicity assays for ballistic aerosols composed of micrometals and nanometals: Respiratory health implications. Int. J. Nanomed..

[15] Leggett R.W. (1997). A model of the distribution and retention of tungsten in the human body. Sci. Total Environ..

[16] Lemus R., Venezia C.F. (2015). An update to the toxicological profile for water-soluble and sparingly soluble tungsten substances. Crit. Rev. Toxicol..

[17] Upadhyay S., Palmberg L. (2018). Air-Liquid Interface: Relevant In Vitro Models for Investigating Air Pollutant-Induced Pulmonary Toxicity. Toxicol. Sci..

[18] Joris F., Manshian B.B., Peynshaert K., De Smedt S.C., Braeckmans K., Soenen S.J. (2013). Assessing nanoparticle toxicity in cell-based assays: Influence of cell culture parameters and optimized models for bridging the in vitro-in vivo gap. Chem. Soc. Rev..

[19] BeruBe K., Prytherch Z., Job C., Hughes T. (2010). Human primary bronchial lung cell constructs: The new respiratory models. Toxicology.

[20] Constant S., Huang S., Derouette J.P., Wiszniewski L. (2008). MucilAir: A novel in vitro human 3D airway epithelium model for assessing the potential hazard of nanoparticles and chemical compounds. Toxicol. Lett..

[21] Dine S., Aid S., Ouaras K., Malard V., Odorico M., Herlin-Boime N., Habert A., Gerbil-Margueron A., Grisolia C., Chene J. (2015). Synthesis of tungsten nanopowders: Comparison of milling, SHS, MASHS and milling-induced chemical processes. Adv. Powder Technol..

[22] Sanles Sobrido M., Bernard E., Angeletti B., Malard V., Georges I., Chaurand P., Uboldi C., Orsière T., Dine S., Vrel D. (2019). Oxidative transformation of Tungsten (W) submicron particles released in aqueous and biological media in case of Tokamak (nuclear fusion) Lost of Vacuum Accident (LOVA). Environ. Sci. Nano.

[23] Grisolia C., Hodille E., Chene J., Garcia-Argote S., Pieters G., El-Kharbachi A., Marchetti L., Martin F., Miserque F., Vrel D. (2015). Tritium absorption and desorption in ITER relevant materials: Comparative study of tungsten dust and massive samples. J. Nucl. Mater..

[24] Ortega R., Bresson C., Darolles C., Gautier C., Roudeau S., Perrin L., Janin M., Floriani M., Aloin V., Carmona A. (2014). Low-solubility particles and a Trojan-horse type mechanism of toxicity: The case of cobalt oxide on human lung cells. Part. Fibre Toxicol..

[25] Stoehr L.C., Endes C., Radauer-Preiml I., Boyles M.S., Casals E., Balog S., Pesch M., Petri-Fink A., Rothen-Rutishauser B., Himly M. (2015). Assessment of a panel of interleukin-8 reporter lung epithelial cell lines to monitor the pro-inflammatory response following zinc oxide nanoparticle exposure under different cell culture conditions. Part. Fibre Toxicol..

[26] Dankers A.C.A., Kuper C.F., Boumeester A.J., Fabriek B.O., Kooter I.M., Grollers-Mulderij M., Tromp P., Nelissen I., Zondervan-Van Den Beuken E.K., Vandebriel R.J. (2018). A practical approach to assess inhalation toxicity of metal oxide nanoparticles in vitro. J. Appl. Toxicol..

[27] Laulicht F., Brocato J., Cartularo L., Vaughan J., Wu F., Kluz T., Sun H., Oksuz B.A., Shen S., Peana M. (2015). Tungsten-induced carcinogenesis in human bronchial epithelial cells. Toxicol. Appl. Pharmacol..

[28] Chinde S., Poornachandra Y., Panyala A., Kumari S.I., Yerramsetty S., Adicherla H., Grover P. (2018). Comparative study of cyto- and genotoxic potential with mechanistic insights of tungsten oxide nano- and microparticles in lung carcinoma cells. J. Appl. Toxicol..

[29] Bastian S., Busch W., Kuhnel D., Springer A., Meissner T., Holke R., Scholz S., Iwe M., Pompe W., Gelinsky M. (2009). Toxicity of Tungsten Carbide and Cobalt-Doped Tungsten Carbide Nanoparticles in Mammalian Cells in Vitro. Environ. Health Perspect..

[30] Hoet P.H., Roesems G., Demedts M.G., Nemery B. (2002). Activation of the hexose monophosphate shunt in rat type II pneumocytes as an early marker of oxidative stress caused by cobalt particles. Arch. Toxicol..

[31] Kasten U., Mullenders L.H.F., Hartwig A. (1997). Cobalt(II) inhibits the incision and the polymerization step of nucleotide excision repair in human fibroblasts. Mutat. Res. DNA Repair.

[32] Paget V., Moche H., Kortulewski T., Grall R., Irbah L., Nesslany F., Chevillard S. (2015). Human cell line-dependent WC-Co nanoparticle cytotoxicity and genotoxicity: A key role of ROS production. Toxicol. Sci..

[33] Nilsson H.E., Dragomir A., Lazorova L., Johannesson M., Roomans G.M. (2010). CFTR and tight junctions in cultured bronchial epithelial cells. Exp. Mol. Pathol..

[34] Huang S., Wiszniewski L., Constant S., Roggen E. (2013). Potential of in vitro reconstituted 3D human airway epithelia (MucilAir) to assess respiratory sensitizers. Toxicol. In Vitro.

[35] Stefaniak A.B. (2010). Persistence of tungsten oxide particle/fiber mixtures in artificial human lung fluids. Part. Fibre Toxicol..

